# Adaptation of *Echinococcus Multilocularis* to Oxidative Stress Depends on EmPDK‐Mediated Metabolic Reprogramming

**DOI:** 10.1002/advs.76829

**Published:** 2026-08-13

**Authors:** Huijuan Wang, Zhijian Xu, Ye Tian, Yao Zhang, Zhe Cheng, Yanhai Wang

**Affiliations:** ^1^ State Key Laboratory of Cellular Stress Biology School of Life Sciences Xiamen University Xiamen Fujian China; ^2^ Parasitology Research Laboratory School of Life Sciences Xiamen University Xiamen Fujian China; ^3^ School of Nursing Guangzhou Xinhua University Guangzhou China

**Keywords:** *Echinococcus multilocularis*, metabolic reprogramming, oxidative stress, pyruvate dehydrogenase kinase

## Abstract

Alveolar echinococcosis (AE), a lethal disease caused by the parasite *Echinococcus multilocularis*, is characterized by tumor‐like growths in a host‐derived oxidative microenvironment. The mechanisms that enable the parasite to adapt its central carbon metabolism to the host's oxidative microenvironment remain poorly understood. Here, we report that oxidative stress serves as a key environmental cue that induces a profound metabolic rewiring. We identified pyruvate dehydrogenase kinase (EmPDK) as the central regulator governing this metabolic adjustment, favoring glycolysis and lactate production over oxidative phosphorylation (OXPHOS). Suppressing EmPDK activity enhances oxidative metabolism and impairs metacestode proliferation, whereas augmenting its function (via H_2_O_2_‐induced upregulation) promotes glycolysis and growth. Reactive oxygen species (ROS) drive this metabolic reprogramming through the transcription factor EmHIF1α, forming an axis of ROS/EmHIF1α/EmPDK that modulates parasite metabolic plasticity. Our findings define EmPDK as a critical mediator of oxidative stress adaptation in *E. multilocularis* and support its further exploration as a candidate target for AE intervention.

## Graphical abstract

Metabolic adaption presents a vital survival strategy for *E. multilocularis*. This study identifies EmPDK as a central metabolic regulator governing glucose metabolic reprogramming. Oxidative stress activates EmHIF1α to induce EmPDK expression. Mechanistic characterization reveals this ROS/EmHIF1α/EmPDK axis drives glycolytic reprogramming to sustain invasive larval growth of the parasite.

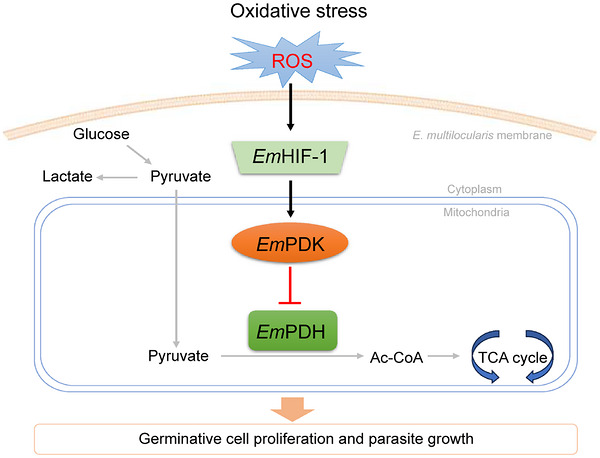

## 1 Introduction

Parasitic existence is the ultimate result of prolonged adaptation and co‐evolution. Compared to their free‐living counterparts, parasites undergo profound morphological and physiological alterations due to their long‐term adaptation to the host environment. Tapeworms, as quintessential obligate parasites, exhibit highly degenerated digestive organs, extremely well‐developed reproductive organs, and structures like suckers that evolved for attachment to the host [1]. Tapeworm eggs or larvae must withstand environmental stressors and develop into infectious stages within intermediate hosts. This process demands high environmental resistance and developmental plasticity, which are critical aspects of their successful parasitic adaptation [2]. Furthermore, to counteract host immune attacks, particularly in nutrient‐competitive microenvironments such as the intestine and liver, parasites must evolve efficient metabolic adaptation strategies to secure energy and survival advantages [3, 4].

Alveolar echinococcosis (AE), a lethal helminthic disease caused by the larval stage of the tapeworm *Echinococcus multilocularis*, is characterized by infiltrative, tumor‐like growth in the liver, posing a substantial public health burden [5, 6, 7]. The metacestode of *E. multilocularis* exhibits aggressive expansion and provokes sustained host immune responses [4, 8, 9, 10]. The infiltrative growth sustains a reactive oxygen species (ROS)‐enriched microenvironment at the host‐parasite interface, generated through sustained immune respiratory bursts [11]. Consequently, the infiltrative growth creates a dynamic, heterogeneous oxidative niche at the invasive front, where tissue damage and chronic inflammation maintain elevated ROS levels [12]. Managing this oxidative stress is therefore not merely a defensive necessity but a prerequisite for the parasite's metabolic homeostasis and sustained growth. Our previous findings demonstrate that the metacestode is highly adapted to oxidative stress [11]. We observed significant ROS accumulation around the hepatic lesions in infected mice. Crucially, experimental reduction of ROS levels decreased the parasite burden and impaired the proliferation of germinative cells, a population of adult stem cells analogous to planarian neoblasts that represent the sole proliferative and pluripotent cell type within the metacestode. Intriguingly, elevated ROS levels actually promoted the growth of *E. multilocularis*. These findings compel a central, unresolved question: through what molecular mechanisms does the parasite not only survival but also thrive within the hostile oxidative microenvironment of host?

Metabolic reprogramming is a core adaptive strategy that supports cellular bioenergetic and biosynthetic demands under stress [13, 14, 15, 16, 17, 18, 19]. A hallmark is the Warburg effect, whereby aerobic glycolysis is favored over oxidative phosphorylation for rapid ATP and precursor generation [18, 19]. Crucially, ROS act as key signaling molecules driving these metabolic changes by regulating mitochondrial function, HIF stability, glycolytic flux, and redox signaling through α‐ketoglutarate and NAD^+^/NADH [20, 21, 22, 23, 24]. This reciprocal crosstalk between oxidative stress and central carbon metabolism constitutes a core adaptive circuit in diverse pathological contexts, including infection and cancer [25, 26, 27].

For parasitic platyhelminthes, encompassing tapeworms and flukes, adaptive regulation of energy metabolism is essential for establishing and maintaining infection [28]. While these parasites possess functional lipid and amino acid metabolic pathways, these systems are often streamlined. Parasitic platyhelminthes generally lack de novo synthesis capabilities for of fatty acids and cholesterol, instead scavenging host lipids [29, 30]. Similarly, their capacity for amino acid biosynthesis is markedly reduced or entirely absent [29, 31]. In contrast, glucose serves as the dominant carbon and energy source [32]. Studies have shown that tapeworms and flukes actively acquire host glucose, exhibit elevated expression of key glycolytic enzymes [33], and require glucose for survival in vitro [32, 34]. However, current understanding remains largely phenomenological, focused primarily on pathway annotation and glucose‐auxotrophic phenotypes. The molecular mechanisms by which these pathogens perceive host environmental cues, such as oxidative stress or immune pressure, and transduce them to dynamically regulate central carbohydrate metabolism remain elusive.

Like other cestodes, *E. multilocularis* lacks a digestive tract and depends on glucose absorbed from the host across its syncytial tegument [35, 36]. Transcriptomic and biochemical studies suggest that the parasite exhibits metabolic flexibility, analogous to the Warburg effect, characterized by sustaining high glycolytic flux concurrent with functional oxidative phosphorylation [31]. This metabolic plasticity may serve as a key adaptation in response to various host nutrients and environment cues, including the host‐derived ROS. However, the regulatory mechanisms governing this metabolic shift remain poorly defined.

In mammals, the pyruvate dehydrogenase kinase (PDK)/pyruvate dehydrogenase (PDH) axis regulates pyruvate flux into the tricarboxylic acid (TCA) cycle toward oxidative metabolism or lactate production [37, 38]. In this study, we identify the homologous genes *EmPDK* and *EmPDH* in *E. multilocularis*, and provide evidence that EmPDK serves as an important downstream effector of ROS signaling, integrating stress cues to drive glycolytic metabolism and sustain parasite proliferation. These findings establish the ROS/EmHIF1α/EmPDK axis as a one key adaptive strategy mediating metabolic adaptation under oxidative stress. We therefore hypothesize that targeting this regulatory module may represent a promising interventional approach to impair parasite survival and proliferation. Accordingly, this study aims to characterize the functional role of this axis and to explore its potential as a regulatory node for future antiparasitic interventions.

## 2 Results

### 2.1 Oxidative Stress Induces Metabolic Reprogramming in *E. multilocularis*, Diverting Glucose Flux From OXPHOS to Glycolysis

The host liver provides an oxidative microenvironment pivotal for both parasite clearance and disease progression. We previously observed that moderate levels of ROS promote metacestode growth and germinative cell proliferation [11]. This implying that the parasite may develop adaptive mechanisms to tolerate and utilize oxidative stress rather than merely resist it. To explore how oxidative stress adaptation affects the metabolism of *E. multilocularis*, the in vitro‐cultivated metacestode vesicles were treated with hydrogen peroxide (H_2_O_2_), followed by metabolomics analysis using liquid chromatography‐mass spectrometry. Orthogonal partial least squares‐discriminant analysis (OPLS‐DA) showed evident metabolic separation between the control and H_2_O_2_‐treated groups (Figure S1A), and robust model parameters (R^2^Y = 1.0, Q^2^ > 0.9) indicated distinct overall metabolic alterations induced by oxidative stress (Figure S1B).

**FIGURE 1:**
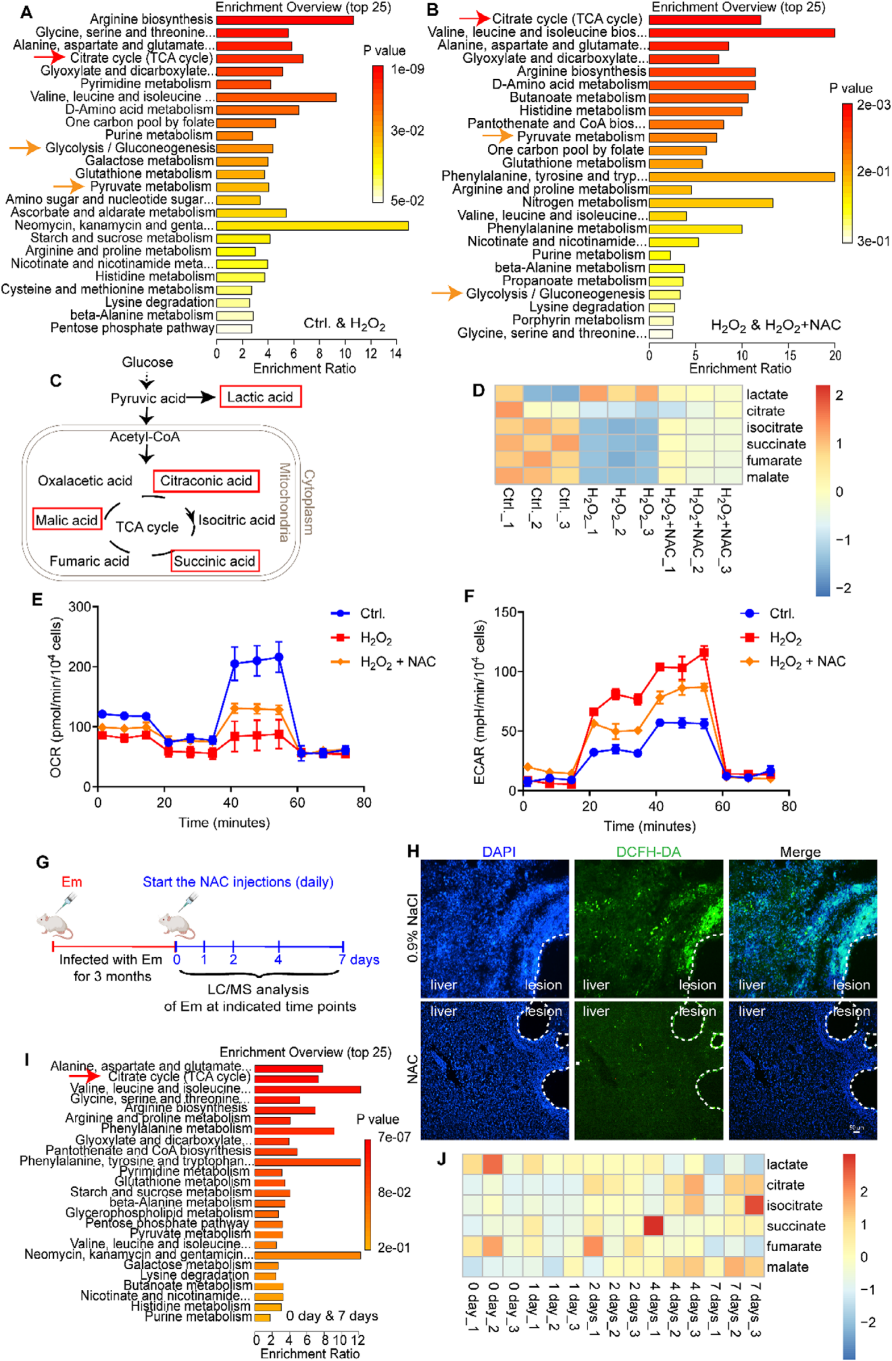
Oxidative stress shifts glucose metabolism from the OXPHOS toward glycolysis in *E. multilocularis. (*A, B, and D). Treatment of metacestode vesicles with H_2_O_2_ (induced oxidative stress) and NAC (ROS scavenging) for 4 h. (A, B) KEGG pathway enrichment of exploratory screened metabolites. Pathway statistics were FDR‐corrected for multiple testing (red arrow: TCA cycle; orange arrows: pyruvate metabolism, glycolysis/gluconeogenesis). (C) Schematic diagram of central glucose metabolic pathways, with key TCA cycle intermediates and lactate highlighted in red. (D) Heatmap of TCA cycle–related metabolites. Metabolite profiles are derived from exploratory screening. (E, F) Time‐course profiles of OCR and ECAR in *E. multilocularis* metacestode vesicles under control, H_2_O_2_, and H_2_O_2_ + NAC treatment. Data are presented as mean ± SD (*n* = 3 technical replicates per group). (G) Schematic representation of the in vivo NAC treatment regimen and subsequent sample processing workflow for *E. multilocularis*‐infected mice. (H) NAC scavenges ROS around parasitic lesions. (I) KEGG pathway enrichment of exploratory screening metabolites from in vivo metacestode lesions after NAC treatment for 7 days. Pathway statistics were FDR‐corrected (red arrow: TCA cycle). (J) Heatmap of TCA cycle–related metabolites after NAC treatment at the indicated time points in in vivo lesions. Metabolite profiles are derived from exploratory screening.

Exploratory screening yielded 146 candidate differential metabolites (Table S1.1, Figure S1C,D). FDR‐corrected KEGG enrichment analysis indicated prominent perturbations of core energy metabolic pathways, including the tricarboxylic acid (TCA) cycle, glycolysis, and pyruvate metabolism, compared to the untreated control (Table S2.1 and Figure 1A). Focusing on​ central energy metabolism, we found that H_2_O_2_ treatment decreased the levels of key TCA cycle intermediates (citrate, isocitrate, succinate, malate, and oxaloacetate) as preliminary trends, while markedly increasing lactate, the end product of glycolysis (Figure 1C,D). Seahorse extracellular flux (XF) metabolic analyses demonstrated that oxidative stress exposure was accompanied by significantly decreased oxygen consumption rate (OCR), indicative of suppressed oxidative phosphorylation, and a marked elevation in extracellular acidification rate (ECAR), consistent with enhanced glycolytic activity in metacestode vesicles (Figure 1E,F). These results suggest that oxidative stress prompts a metabolic shift in glucose utilization, from oxidative phosphorylation (OXPHOS) toward glycolysis in the parasite.

To confirm the regulatory role of ROS in the metabolic shift, the vesicles were co‐treated with H_2_O_2_ and the ROS scavenger N‐acetylcysteine (NAC). Metabolomic profiling showed distinct metabolic signatures distinguishing H_2_O_2_ + NAC group from the H_2_O_2_ only group (Figure S1E,F), with 119 candidate differential metabolites (Table S1.2, Figure S1G,H). KEGG enrichment analysis showed significant enrichment of the TCA cycle, glycolysis, and pyruvate metabolism pathways (Table S2.2 and Figure 1B). Notably, ROS scavenging reversed H_2_O_2_‐triggered metabolic shift: preliminary metabolite trends showed elevated TCA intermediate abundance and reduced lactate accumulation (Figure 1D). Seahorse XF assays further confirmed restored OXPHOS (higher OCR) and suppressed glycolysis (lower ECAR) upon ROS scavenging (Figure 1E,F). Furthermore, direct comparison among the control, NAC‐only, and H_2_O_2_+NAC groups revealed that NAC treatment alone caused no significant alterations in TCA cycle metabolites relative to the control group. In contrast, NAC effectively restored the H_2_O_2_‐induced reduction in TCA cycle metabolites toward control levels (Tables S1.3, S1.4, S2.3, and S2.4, Figure S2). These data indicate that exogenous oxidative stress is associated with glucose metabolism reprogramming in *E. multilocularis*, characterized by reduced TCA cycle/OXPHOS flux and enhanced glycolysis, this metabolic rewiring is reversed upon ROS scavenging by NAC.

To explore whether this ROS‐ associated metabolic plasticity exists in vivo, a murine hepatic infection model was employed. OPLS‐DA revealed a clear separation between the metabolic profiles of NAC‐treated and control lesions (Table S1.5, Figure S3A–L). KEGG enrichment analysis of exploratory candidate metabolites revealed that the TCA cycle was the pathway most significantly altered by NAC treatment, suggesting the restoration of oxidative metabolic flux. As preliminary trends, subsequent analysis showed increased levels of key TCA cycle intermediates (e.g., citrate, isocitrate, succinate and malate), along with a reduction in lactate levels (Table S2.5, Figure 1G–J, Figure S3M–O). Collectively, these in vivo and in vitro findings suggest that oxidative stress triggers a metabolic shift from OXPHOS to glycolysis in *E. multilocularis*, and this stress‐induced metabolic reprogramming is recapitulated in vivo.

### 2.2 EmPDK is a Key Regulator of Glucose Metabolism in *E. multilocularis*

**FIGURE 2:**
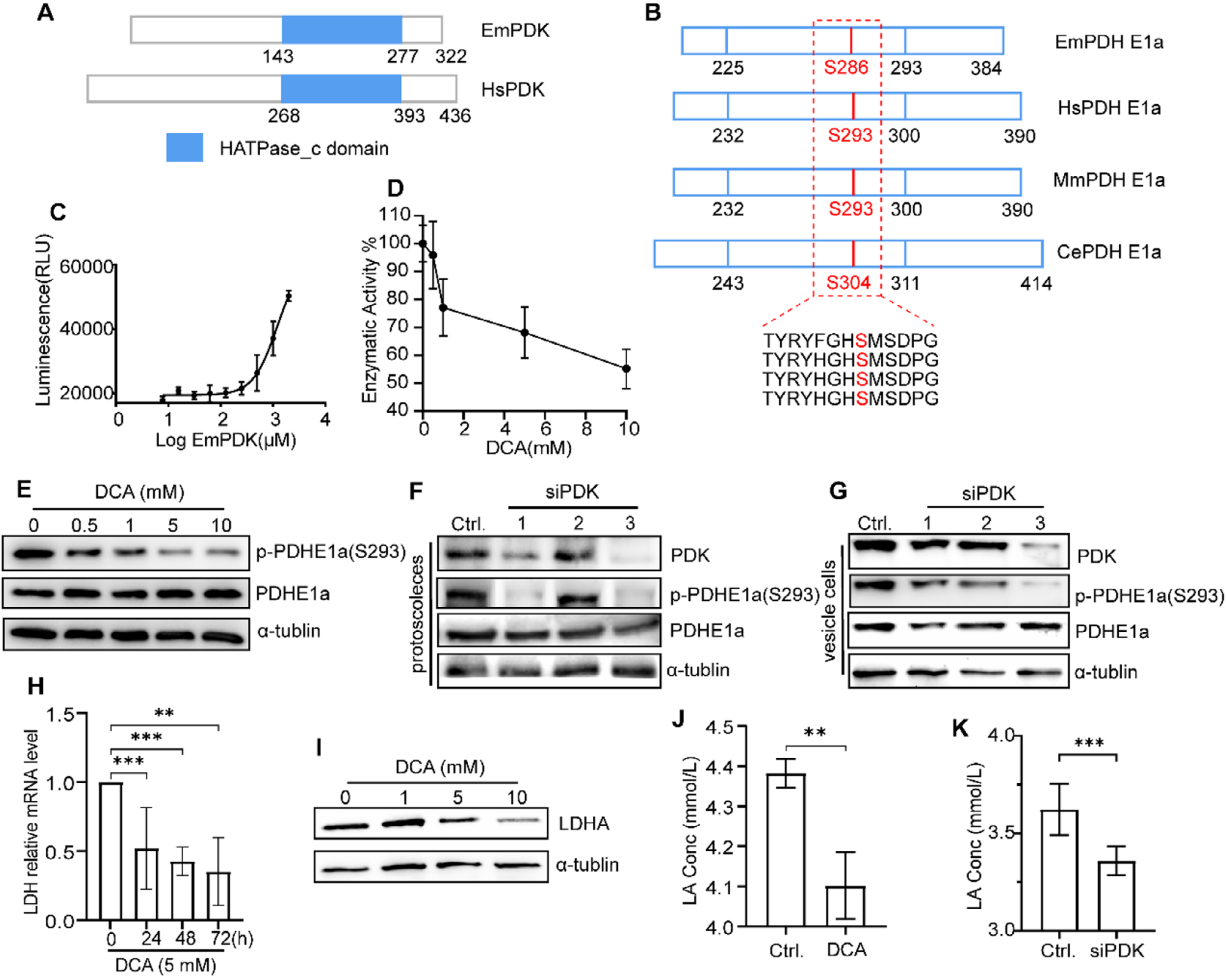
EmPDK acts as a metabolic regulator that diverts the flux of glucose metabolism in *E. multilocularis*. (A) Comparison of the PDK structure in *E. multilocularis* with its corresponding homologs in humans. (B) Sequence alignment of the serine phosphorylation site within the PDHE1α domain of *E. multilocularis* is provided for comparative analysis, including proteins from humans (*Homo sapiens*, Hs), mice (*Mus musculus*, Mm), and *Caenorhabditis elegans* (Ce). (C) EmPDK enzymatic activity. x axis, EmPDK concentration; y axis, relative luminescence units; nonlinear fit. (D) Effect of DCA on EmPDK kinase activity in vitro. Activity is expressed as a percentage of the uninhibited control (mean ± SD; *n*  =  3 biological replicates). (E) Immunoblot analysis of p‐EmPDHE1α expression in metacestode vesicles treated with DCA for 4 h. (F, G) Immunoblot analysis of EmPDK and p‐EmPDHE1α in protoscoleces and in primary cells isolated from metacestode vesicles following siRNA‐mediated EmPDK knockdown. Lanes 1–3 under “siPDK” represent three independent siRNA sequences targeting the *EmPDK* gene. (H) *EmLDHA* mRNA levels in metacestode vesicles treated with DCA. (I) Immunoblot analysis of EmLDHA expression in metacestode vesicles treated with DCA for 4 h. (J, K) Lactic acid content in metacestode vesicles with DCA and in protoscoleces following siRNA‐mediated EmPDK knockdown. *E. multilocularis* metacestode vesicles or protoscoleces were analyzed in panels (H), (J) and (K); n = 3 biological replicates, each with three technical replicates. Data are mean ± SD; One‐way ANOVA with Tukey's HSD post‐hoc test. *p < 0.05, **p < 0.01, ***p < 0.001.

Through homology searches, we identified the *E. multilocularis* homologs of PDK and PDHE1α, named EmPDK and EmPDHE1α respectively. EmPDK and EmPDHE1α exhibit domain structures similar to their mammalian counterparts, and EmPDHE1α has three conserved phosphorylation sites in which the highest similarity of amino acid sequence is found near Ser293. We cloned the corresponding genes and generated specific polyclonal antibodies for subsequent detection and functional analysis (Figure 2A,B, Figure S4). An in vitro kinase assay confirmed the functional activity of EmPDK and demonstrated that its kinase activity could be inhibited by the PDK inhibitor dichloroacetate (DCA) (Figure 2C,D).

To assess the functional role of EmPDK in glucose metabolism, we treated the parasite with either DCA or subjected them to EmPDK knockdown via RNA interference (siRNA). Both approaches significantly reduced phosphorylation of EmPDHE1α at Ser293 (Figure 2E–G), confirming effective suppression of EmPDK activity. This inhibition resulted in a marked downregulation of glycolytic flux, as demonstrated by the decreased mRNA and protein expression of lactate dehydrogenase (EmLDHA) (Figure 2H,I) and a significant reduction in lactate accumulation (Figure 2J,K). These results indicate that EmPDK functions as a critical metabolic regulator that promotes glycolytic flux by inactivating EmPDHE1α via phosphorylation, thereby diverting glucose metabolism away from mitochondrial OXPHOS and toward lactate fermentation.

### 2.3 EmPDK is Essential for Germinative Cell Proliferation and Parasite Growth

**FIGURE 3:**
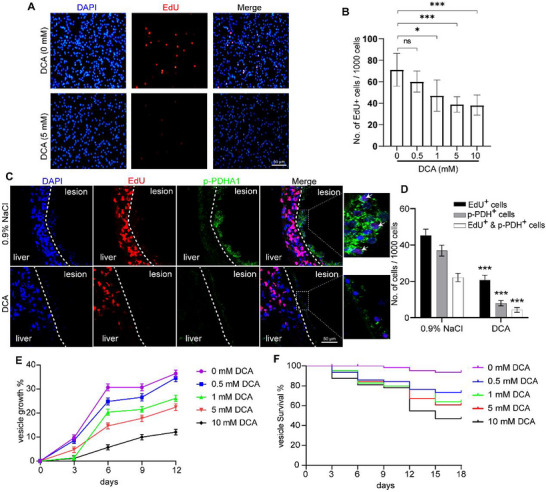
Inhibition of EmPDK impairs proliferation and growth rate of *E. multilocularis*. (A) Germinative cells in metacestode vesicles treated with DCA for 48 h, blue: DAPI; red: EdU. (B) Quantification of EdU^+^ cells per 1000 cells from the experiment in (A). (C) Immunofluorescence staining of liver lesion sections from infected mice treated with DCA and EdU. DAPI (blue), EdU (red), and anti‐p‐EmPDH E1α (green). (D) Quantification of EdU^+^, p‐EmPDH^+^, and EdU^+^/p‐EmPDH^+^ double‐positive cells per 1000 cells in metacestode vesicles isolated from liver lesions treated with DCA for 2 weeks. (E, F) Growth rate and survival rate of in vitro cultured metacestode vesicles (mean ± SD; *n* = 3 biological replicates). Panels B and D show EdU^+^ proliferative cell counts in *E. multilocularis* lesions. Single metacestode vesicle (B, *n* = 10 biological replicates); Infected mouse (D, *n* = 7 biological replicates). Data are mean ± SD. One‐way ANOVA with Tukey's HSD post‐hoc test. *p < 0.05, **p < 0.01, ***p < 0.001; ns = not significant.

The growth of *E. multilocularis* metacestode larvae is driven by the germinative cells, a population of neoblast‐like adult stem cells. We next asked whether PDK‐mediated metabolic alterations are involved in regulating germinative cell proliferation. EdU labeling assays in metacestode vesicles showed that EmPDK inhibition significantly reduced the proportion of EdU^+^ cells (Figure 3A,B). A detailed temporal analysis further demonstrated that short‐term EmPDK suppression did not markedly alter the overall abundance of EdU^+^ or p‐EmPDHE1α (Ser293)^+^ cells. However, within the proliferating EdU^+^ population, the fraction of cells positive for phosphorylated EmPDHE1α was significantly reduced (Figure S5A–F). Prolonged inhibition resulted in a pronounced decrease across all metrics: total EdU^+^ cells, p‐EmPDHE1α^+^ cells, and notably, the prevalence of p‐EmPDHE1α within the proliferative compartment (Figure S5H–K). The decline in EdU incorporation lagged behind the rapid loss of phospho‐EmPDHE1α signal, an effect that was especially stark within the EdU^+^ cell populations (Figure S5G).

In the infected mice, DCA treatment also significantly reduced EdU labeling in the parasite lesions (Figure 3C,D). Concordantly, substantial decreases were observed in both p‐EmPDHE1α^+^ cells and the proportion of p‐EmPDHE1α^+^ cells among the remaining EdU^+^ germinative cells (Figure S5L–O). Together, these findings suggest that EmPDK‐mediated metabolic reprogramming is essential for sustaining germinative cell proliferation.

We further evaluated the organismal phenotypic consequences of EmPDK inhibition. Dose‐ and time‐dependent DCA treatment markedly impaired metacestode vesicle growth in vitro (Figure 3E). Notably, sustained EmPDK inhibition led to a progressive decline in viability (Figure 3F).

Together, these results demonstrated that EmPDK‐mediated reprogramming of glucose metabolism, achieved through phosphorylation of EmPDHE1α, is essential for sustaining germinative cell proliferation and larval growth of the parasite.

### 2.4 Oxidative Stress‐induced Metabolic Reprogramming via EmPDK Promotes Proliferation and Adaptation in *E. multilocularis*

**FIGURE 4:**
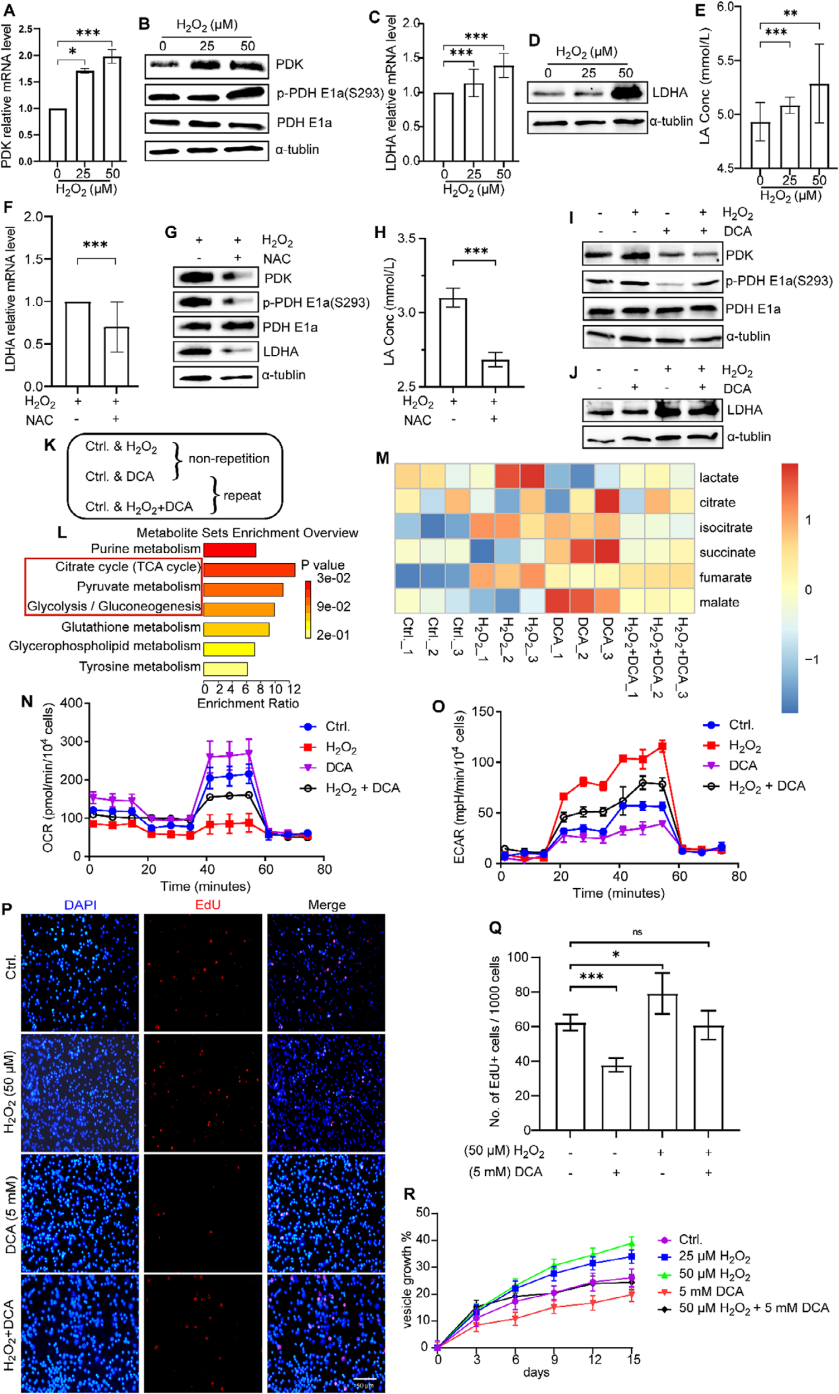
EmPDK mediates the glucose metabolic reprogramming induced by oxidative stress in *E. multilocularis*. (A–E) Treatment of metacestode vesicles with H_2_O_2_ for 24 h. (A) *EmPDK* mRNA levels were measured by RT‐qPCR. (B) Immunoblot analysis of EmPDK and p‐EmPDHE1α expression. (C) *EmLDHA* mRNA levels were measured by RT‐qPCR. (D) Immunoblot analysis of EmLDHA expression. (E) Testing for Lactic acid content. (F–H) After co‐treatment with H_2_O_2_ and NAC. (F) *EmLDHA* mRNA levels were measured by RT‐qPCR. (G) Immunoblot analysis of EmPDK, p‐EmPDH E1α, and EmLDHA expression. (H) Testing for Lactic acid content. (I, J) Immunoblot analysis of EmPDK, p‐EmPDH E1α, and EmLDHA in vesicles treated with: (1) control, (2) H_2_O_2_ for 24 h, (3) DCA for 4 h, and (4) combined H_2_O_2_ and DCA treatment. (K) Workflow for screening shared differential metabolites across the three treatment groups (related to L). Briefly, upregulated and downregulated metabolites were separately analyzed in the first two comparisons to exclude intra‐group overlapping hits. The remaining distinct metabolites were then intersected with those from the third comparison to identify the final shared differential metabolites. (L) KEGG enrichment analysis of differentially abundant metabolites identified by exploratory screening, including intersection and secondary enrichment across all three experimental groups. Pathway significance was determined using FDR‐corrected statistics. (M) Heatmap of TCA cycle‐related metabolites and lactate levels in metacestodes under control, H_2_O_2_, DCA, and H_2_O_2_ + DCA conditions. Metabolite profiles are derived from exploratory screening. (N, O) Time‐course profiles of OCR and ECAR in *E. multilocularis* metacestode vesicles subjected to control, H_2_O_2_, DCA, and H_2_O_2_ + DCA treatments. Data are presented as mean ± SD (n = 3 technical replicates per group). (P) Immunofluorescence staining of metacestode vesicles following single or combined treatment with H_2_O_2_ and DCA for 24 h. DAPI (blue), EdU (red). (Q) Quantification of EdU^+^ cells from (M). (R) Growth rate of in vitro‐cultured metacestode vesicles treated with H_2_O_2_ and DCA, as well as the co‐treatment group of H_2_O_2_ and DCA. Data in (A, C, E, F, H, Q, R) are presented as mean ± SD; Panels A, C, E, F, H: metacestode vesicle samples; n = 3 biological replicates, three technical replicates per sample. Panel Q: single metacestode vesicle (*n* = 7 biological replicates). All multi‐group panels were analyzed using one‐way ANOVA followed by Tukey's HSD post‐hoc test. Panel R: culture well holding 20–50 metacestode vesicles (*n* = 3 replicate wells). *p < 0.05, **p < 0.01, ***p < 0.001; ns = not significant.

To investigate whether oxidative stress reprograms the metabolic network of *E. multilocularis* via EmPDK, metacestode vesicles were exposed to H_2_O_2_. The treatment significantly increased EmPDK expression at both mRNA and protein levels and enhanced phosphorylation of its downstream substrate EmPDHE1α at Ser293 (Figure 4A,B), indicating an activation of the EmPDK signaling axis. Consequently, elevated EmLDHA expressions and increased lactate accumulations were observed upon H_2_O_2_ exposure (Figure 4C–E), demonstrating a metabolic shift from TCA cycle/OXPHOS toward aerobic glycolysis. While co‐treatment with H_2_O_2_ and NAC suppressed the H_2_O_2_‐induced upregulation of EmLDHA mRNA, EmPDK protein, phospho‐EmPDHE1α (Ser293), EmLDHA protein, and lactate production (Figure 4F–H), thereby confirming the impacts of oxidative stress on the glucose metabolism of the parasite.

To assess the functional contribution of EmPDK, vesicles were co‐treated with H_2_O_2_ and DCA. As shown in Figure 4I,J, H_2_O_2_ treatment alone upregulated EmPDK and EmLDHA expression. In contrast, DCA alone suppressed phospho‐EmPDHE1α (S293) and reduced EmLDHA levels. Notably, co‐treatment with H_2_O_2_ and DCA partially antagonized the H_2_O_2_‐induced upregulation of EmLDHA. These results suggest that oxidative stress modulates glucose metabolism flux in part through EmPDK activity, thereby redirecting pyruvate toward lactate production. Collectively, these findings elucidate one contributory mechanism by which oxidative stress, via EmPDK activation, promotes glycolytic reprogramming in *E. multilocularis*, highlighting a direct molecular link between host environmental stress and parasite metabolic adaptation.

To further define the role of EmPDK in the metabolic response of *E. multilocularis* under oxidative stress, we performed metabolomic profiling of metacestode vesicles treated with DCA. Exploratory screening identified candidate differential metabolites, and FDR‐corrected KEGG enrichment analysis revealed significant overrepresentation of core energy metabolism pathways, including the TCA cycle, glycolysis, and pyruvate metabolism (Tables S1.6 and S2.6, Figure S6A–E). This enrichment was conserved in both H_2_O_2_‐treated and H_2_O_2_+DCA co‐treated groups (Table S2.7, Figure S6F,G). Intersection analysis and secondary enrichment analysis of exploratory candidate metabolites across all three experimental groups indicate that more than half of the significantly enriched pathways are related to carbohydrate metabolism (Table S2.8, Figure 4K,L). Focusing on key TCA cycle metabolites (Figure 4M), preliminary metabolite trends revealed three principal observations: First, H_2_O_2_ treatment reduced levels of intermediates, including citrate, succinate and malate, while promoting lactate accumulation, indicating a shift toward glycolytic metabolism under oxidative stress. Second, DCA treatment alone produced the opposite effect, trending toward increased TCA cycle intermediates and decreased lactate. Third, in the H_2_O_2_+DCA co‐treatment group, DCA partially counteracted the H_2_O_2_‐driven metabolic alterations, restoring TCA cycle intermediate abundance and attenuating lactate accumulation. XF analyses were performed on DCA‐treated metacestode vesicles, revealing that DCA treatment led to increased OCR, indicative of enhanced oxidative phosphorylation, and suppressed ECAR, which is consistent with the ability of DCA to antagonize the glycolytic shift induced by oxidative stress (Figure 4N,O). These results suggest that oxidative stress triggers EmPDK‐dependent metabolic reprogramming in *E. multilocularis*, enabling a reversible transition between oxidative and glycol metabolic states that promotes adaptation to redox stress.

We also found that, along with the enhanced glycolytic flux, H_2_O_2_ treatment promoted germinative cell proliferation and vesicle growth, an effect that was significantly reduced by the PDK inhibitor DCA and eliminated by combined H_2_O_2_ and DCA treatment (Figure 4P–R). While we have demonstrated that scavenging ROS [11] or inhibiting EmPDK activity attenuated this pro‐proliferative effect (Figure 3) in vivo. These results are consistent with the idea that the ROS/EmPDK‐driven shift to a glycolytic state supports the invasive growth and host adaptation of *E. multilocularis* by providing necessary bioenergetic and biosynthetic precursors.

### 2.5 EmHIF1α is Involved in Oxidative Stress–Induced Metabolic Reprogramming via EmPDK

**FIGURE 5:**
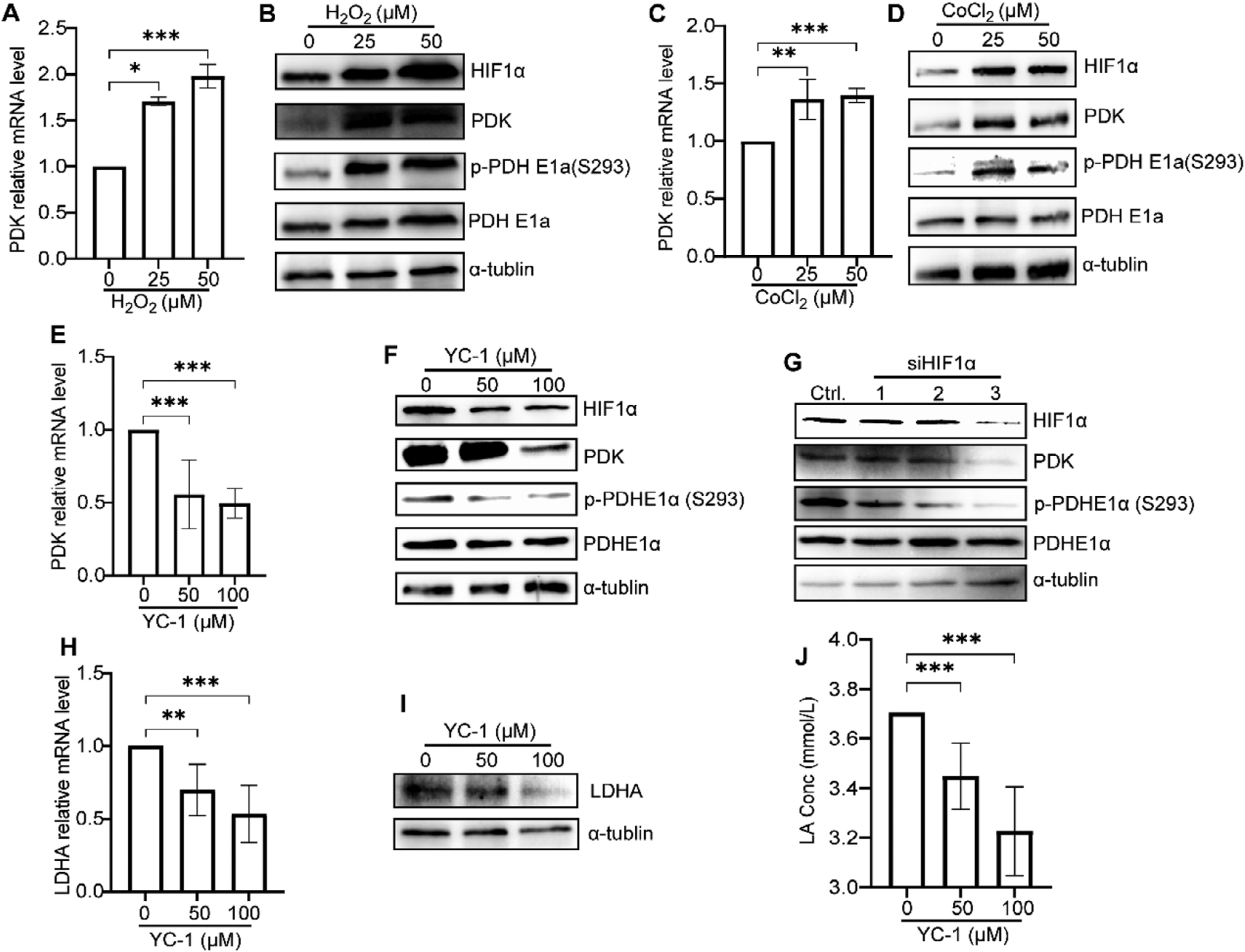
Oxidative stress signals through EmHIF1α to activate EmPDK and promote glycolysis in *E. multilocularis*. (A‐F and H‐J) Treatment of metacestode vesicles with H_2_O_2_, CoCl_2_ and YC‐1 for 24 h. (A, C, E) *EmPDK* mRNA levels were measured by RT‐qPCR. (B, D, F) Immunoblot analysis of EmHIF‐1α, EmPDK and p‐EmPDHE1α expression. (G) Immunoblot analysis of EmHIF‐1α, EmPDK, and p‐EmPDHE1α in vesicles transfected with three independent siRNAs targeting *EmHIF‐1α* (lanes 1–3) or a control siRNA. (H) *EmLDHA* mRNA levels were measured by RT‐qPCR. (I) Immunoblot analysis of EmLDHA expression. (J) Testing for Lactic acid content. Data in (A, C, E, H, J) are presented as mean ± SD; Metacestode vesicle samples; n = 3 biological replicates with three technical replicates each. All multi‐group panels were analyzed using one‐way ANOVA followed by Tukey's HSD post‐hoc test. *p < 0.05, **p < 0.01, ***p < 0.001.

We previously identified the HIF‐1α homolog of *E. multilocularis* (EmHIF‐1α) and demonstrated that ROS regulates *E. multilocularis* germinative cell proliferation and larval growth via upregulating EmHIF‐1α expression. In mammals, HIF‐1αdirectly regulates PDK expression through binding to hypoxia‐response elements (HREs) in its promoter. To explore this potential regulatory interaction in parasites, bioinformatic analyses were performed that predicted HREs within the promoter region of *EmPDK*. Dual‐luciferase reporter assays further revealed that EmHIF‐1α could target the EmPDK promoter to activate its transcription (Figure S7). We then treated vesicles with H_2_O_2_ and observed significantly increased levels of both EmPDK mRNA and protein, accompanied by increased phosphorylation of EmPDHE1α (Figure 5A,B). Similar results were observed when vesicles treated with the HIF‐1α inducer CoCl_2_ (Figure 5C,D). EmHIF‐1α knockdown or inhibition with lificiguat (YC‐1) reduced EmPDK expression and diminished EmPDHE1α phosphorylation (Figure 5E–G), indicating that EmHIF‐1α functions as one important positive upstream regulator of EmPDK. Meanwhile, inhibition of EmHIF‐1α resulted in decreased transcription and translation of lactate dehydrogenase (EmLDHA) (Figure 5H,I) and a corresponding reduction in lactate secretion (Figure 5J), indicating an impaired glycolytic flux. Together, these results demonstrate that EmHIF‐1α contributes to a glycolytic shift by upregulating EmPDK, which inactivates EmPDHE1α via phosphorylation and promotes lactate production.

## 3 Discussion

Classical evolutionary theory often posits the environment as a static backdrop to which organisms passively adapt. In contrast, the concept of niche construction presents a dynamic framework where in organisms actively modify their environment through physiology, behavior, and metabolism, thereby altering the very selective pressures acting upon them and establishing a feedback loop of reciprocal influence [39]. Parasitism represents a quintessential manifestation of this process. Parasites establish niches by occupying space within hosts and reshaping local environments through metabolic byproducts and immune modulation [40]. These effects can produce transgenerational impacts, continuously shaping the evolutionary trajectory of parasites. Our findings elucidate a sophisticated adaptive mechanism in *E. multilocularis* whereby the parasite co‐opts host‐derived oxidative stress, transducing it into a metabolic signal that sustains its survival and growth. This exemplifies niche construction, as the parasite actively remodels its environment to reshape selective pressures rather than merely adapting to them. We identified a central signaling axis, ROS‐EmHIF1α‐EmPDK, that functions as a molecular processor, converting a hostile host immune signal (ROS) into instructions for a profound metabolic shift from oxidative phosphorylation to glycolysis (Figure 4). This constitutes not a passive response but an active, orchestrated reprogramming that facilitates parasite persistence within the hostile host milieu.

Oxidative stress is a central battleground in host‐parasite interactions. Immune effector cells deploy a respiratory burst, generating copious ROS to directly eliminate pathogens [41, 42]. Paradoxically, excessive ROS can inflict collateral damage on host tissues, exacerbating inflammation [43, 44]. In response to this potent selective pressure, successful parasites have evolved intricate oxidative stress management systems [45]. Our data indicate that *E. multilocularis* metacestodes employ a proactive strategy: they metabolically reinterpret ROS from a cytotoxic threat into a signaling cue (Figure 1). H_2_O_2_ is a physiological ROS molecule that functions as a key signaling molecule. At low, homeostatic concentrations in the nanomolar range, H_2_O_2_ acts as a second messenger that modulates critical pathways, such as MAPK, NF‐κB, and PI3K/AKT. Under pathological or severe stress conditions, such as during infection or sustained oxidative stress, H_2_O_2_ levels can rise to the micromolar range. These elevated concentrations can overwhelm cellular antioxidant defenses, causing damage, and may activate distinct, high‐threshold signaling responses. This contributes to inflammation and disease progression [46, 47, 48]. Here, we demonstrate that it orchestrates a metabolic shift from OXPHOS to glycolysis and lactate production via the ROS‐EmHIF1α‐EmPDK axis (Figure 5). This reprogramming confers dual adaptive advantages: it attenuates endogenous mitochondrial ROS production, mitigating oxidative damage [20, 23], and enables rapid ATP generation alongside the provision of biosynthetic precursors to support proliferation under stress [18, 19]. Crucially, scavenging ROS with NAC reversed this metabolic shift, confirming ROS as the primary driver [49, 50]. This also emphasizes that the ROS‐triggered signaling pathway (represented by the ROS/EmHIF1α/EmPDK axis) is essential for the parasite's metabolic adaptation and proliferative response to oxidative stress. Therefore, disrupting this specific, high‐threshold ROS signaling pathway may represent a more targeted interventional approach compared with global ROS scavenging [51, 52].

The HIF‐PDK cascade is well established as the dominant pathway mediating ROS‐dependent metabolic adaptation in mammals. Our stepwise validation experiments demonstrate that this axis also contributes to governing ROS‐induced glycolytic reprogramming in *E. multilocularis*. However, ROS can activate multiple alternative signaling cascades that further regulate PDK activity, such as the MAPK and PI3K‐Akt pathways, and this study cannot exclude their potential involvement or crosstalk with the HIF‐PDK axis. As such, whether these pathways mediate ROS‐regulated EmPDK expression or function warrants further exploration.

The evolutionary implications of metabolic plasticity are profound. The observed Warburg‐like effect in *E. multilocularis* metacestode stage mirrors strategies in other parasitic platyhelminths, such as *Schistosoma mansoni*, which highly expresses TCA cycle genes in its free‐living cercarial stage but markedly shifts toward glycolysis with concomitant TCA cycle downregulation upon transitioning to an obligate parasitic adulthood [53, 54, 55, 56]. This convergence suggests a fundamental evolutionary solution to the challenges of parasitism. The conservation of glycolytic dominance, despite its lower ATP yield per glucose molecule, indicates it provides critical non‐energy‐related advantages for parasitic success, including reduced mitochondrial ROS and rapid biosynthetic precursor generation. This supports the hypothesis that metabolic reprogramming is an evolutionary innovation within the Platyhelminthes phylum, embedded within developmental programs to facilitate the colonization of disparate ecological niches.

This study suggests that the metabolic reprogramming of *E. multilocularis* in response to oxidative stress through the ROS‐EmHIF1α‐EmPDK signaling pathway is a highly integrated, adaptive process. This shifts energy production from oxidative phosphorylation to glycolysis, leading to increased production of key metabolic byproducts, notably lactate. The local accumulation of lactate acidifies the microenvironment, thereby suppressing the function of effector immune cells and promoting an immunoregulatory phenotype [57, 58, 59]. Therefore, the parasite's metabolic adaptation to oxidative stress is not merely an energy shift, but an integrated survival strategy that counteracts oxidative damage, mediates immune evasion, supports biosynthesis, and sustains proliferation to favor survival in the hostile host environment. Nevertheless, the role of parasite‐derived lactate in immune modulation remains largely unclear. More refined experiments are needed in future studies to clarify its contribution to host–parasite interactions in AE.

This “metabolism‐immune microenvironment” axis provides a novel pathogenic framework for alveolar echinococcosis. The high recurrence and treatment‐resistant nature of this disease may be partly attributable to this metabolic adaptation, which fuels the relentless proliferation of germinative cells. Notably, our data strongly support that the ROS–EmHIF1α–EmPDK axis contributes to orchestrating glycolytic reprogramming and regulating *E. multilocularis* growth. However, efficient gene overexpression and complementation systems remain undeveloped in flatworms. Owing to these technical constraints, definitive gain‐of‐function validation was not achieved in this study. Further advances in flatworm genetic manipulation will enable rigorous causal verification of this axis in future work. Consequently, targeting the core of this mechanism, for instance, using pharmacological inhibitors of EmPDK such as DCA, represents a promising therapeutic strategy. By compelling the parasite to revert to oxidative metabolism, this approach could simultaneously disrupt energy production, increase vulnerability to oxidative stress, and ameliorate the immunosuppressive niche, thereby compromising parasite survival on multiple fronts. Moreover, the clinical translational potential of this metabolic axis warrants deeper investigation. Future studies based on clinical patient samples are needed to elucidate the correlations between the expression levels of key markers (EmHIF1α, EmPDK, p‐EmPDHE1α, and EmLDHA) and the invasiveness and clinical severity of AE lesions, so as to further validate the clinical implications of our findings.

This study is the first to focus on PDK, a key metabolic checkpoint in the glycolytic pathway of *E. multilocularis*. It systematically elucidates PDK's central role in mediating the parasite's oxidative stress response and glucose metabolism reprogramming. Unlike previous studies on *E. multilocularis* that examined upstream regulatory factors [60, 61, 62] or metabolic pathways in S. mansoni [63, 64, 65, 66], this study targets the regulatory hub at the metabolic execution level. The study reveals that PDK not only executes upstream signals, but also serves as a functional center that directly integrates stress signals, modulates metabolic dynamics, and regulates redox homeostasis (Figure 2). This discovery establishes one direct link in *E. multilocularis* between changes in the activity of specific metabolic enzymes and the functional outcomes of oxidative stress adaptation and energy metabolism remodeling. It provides direct evidence for reverse‐engineering the adaptive strategies that parasites use to cope with environmental stress.

In conclusion, our study delineates a coherent signaling‐to‐metabolite cascade that highlights the dynamic interplay between a parasite and its host. *E. multilocularis* exemplifies how parasites evolve not merely to withstand host defenses but to co‐opt them, harnessing host‐derived signals to drive adaptive metabolic innovations that ensure propagation and survival. This work transcends a static view of host‐parasite interaction, revealing it as a perpetually evolving dialogue mediated by metabolism. It provides a mechanistic framework that bridges host immunology, parasite metabolism, and evolutionary ecology, offering a dynamic view of host‐parasite dialogue.

## 4 Experimental Section/Methods

### 4.1 Ethics Statement

All animal experiments were conducted in strict accordance with China regulations on the protection of experimental animals (Regulations for the Administration of Affairs Concerning Experimental Animals, version from July 18, 2023) and specifically approved by the Institutional Animal Care and Use Committee of Xiamen University, China (Permit Number: 2020–0072).

### 4.2 Parasite and Cell Culture, Reagents, and Growth Assay

The *E. multilocularis* isolate used in this study was derived from the Hulunbeier pasture in Inner Mongolia, China, and has been maintained by serial passage in KM mice supplied by the Xiamen University Laboratory Animal Centre (XMULAC). Metacestode vesiculation was performed using a previously established protocol in HeLa medium (Spiliotis and Brehm, 2009). For vesicle growth assays, healthy vesicles with a diameter of ≤1 mm were manually selected and cultured in 12‐well plates with HeLa medium containing the indicated reagents. Except for the concentration gradients indicated in corresponding Figures, the drug concentrations applied in this study were consistently 50 µm H_2_O_2_ (Sinopharm, China), 5 mm DCA (Selleck, China), and 5 mm NAC (Selleck, China). All vesicles were treated with the above agents for identical durations. Vesicle diameter was measured every three days to assess growth. Quantitative growth analysis was performed based on diameter changes of individual viable vesicles, which were defined as the basic statistical units. Each experimental group contained at least 50 vesicles and was performed in duplicate or triplicate across two to three independent experiments. The following reagents were used: cobalt chloride (Sigma, USA); and YC‐1 (MedChemExpress, USA). HeLa cells were provided by Han's Lab (Xiamen University, China) and maintained in DMEM with 10% FBS at 37°C under 5% CO_2_.

### 4.3 5‐Ethylnyl‐2’‐deoxyuridine (EdU) Labeling

Metacestode vesicles and protoscoleces were incubated with 50 µM of EdU for 4 h and wholemount prepared according to Cheng and colleagues (Cheng et al., 2015). EdU proliferation signals were detected using the BeyoClick EdU Cell Proliferation Kit (Alexa Fluor 594, Beyotime, Shanghai, China). All click‐chemistry reactions were performed at room temperature in the dark, and sample nuclei were counterstained with 1 µg/mL DAPI (Sigma, USA). For the quantification of EdU^+^ cells in metacestode vesicles, 3–5 random microscopic fields per vesicle from 7 to 10 vesicle were captured and the positive cells were manually counted. For the quantification of EdU^+^ cells in protoscoleces, the protoscoleces were photographed and the image with the largest number of EdU^+^ cells was taken for counting. At least 2 labeling experiments were performed and analyzed for each control and treatment group.

### 4.4 Immunofluorescence Staining

All animal experiments were performed using 8–10‐week‐old female Kunming mice, with all protocols pre‐approved by the Institutional Animal Care and Use Committee of Xiamen University. Parasite‐infected mice were randomly divided into three groups (DCA, NAC, and saline control), with seven mice per group (*n* = 7). Mice received daily intraperitoneal injections for two consecutive weeks: 100 mg/kg DCA for the DCA group, 150 mg/kg NAC (ROS scavenger) for the NAC group, and equal volumes of sterile saline for the control group with identical treatment schedules. For in vivo proliferation labeling, mice were intraperitoneally injected with 50 mg/kg EdU daily for one week before sample collection. For the frozen sections, liver tissue with lesions were excised form infected mice and enbedded in OCT (Optimal Cutting Temperature, Sakara, United States). Serial sections were performed with Leica cryostat (Leica Biosystems, Germany) and mounted onto slides. EdU were determined by the BeyoClick EdU Cell Proliferation Kit with Alexa Fluor 594 (Beyotime, China).

For the quantification of EdU^+^ cells in mice, 3–5 random non‐overlapping microscopic fields per mouse were captured and the positive cells were counted. Positive staining signals and proliferative cells in each field were manually quantified, and average values were calculated for statistical analysis. All animal intervention and staining experiments were repeated in no fewer than three independent biological trials.

### 4.5 Sample Preparation and Metabolomic Analysis of Metacestode Vesicles

In vitro, following a 4‐h treatment with 50 µM H_2_O_2_, 5 mM DCA, 5 mM NAC, 50 µM H_2_O_2_ + 5 mM NAC, or 50 µM H_2_O_2_ + 5 mM DCA, vesicles were collected, mechanically disrupted using a 1‐mL pipette tip, and centrifuged at 500 g for 10 min at 4 °C to separate vesicle cells (pellet) from vesicle fluid (supernatant). The cell pellet was washed 2–3 times with 5–10 mL PBS, centrifuged again, and resuspended in 1 mL PBS. After transfer to a 1.5‐mL tube and centrifugation, the cell pellet was extracted with 1 mL of ice‐cold extraction solvent (methanol: acetonitrile: water = 2:2:1, v/v/v). The suspension was vortexed for 30 s, subjected to ice‐bath ultrasonication for 10 min, and then frozen in liquid nitrogen and thawed on ice, followed by another 10‐min ice‐bath sonication; this freeze‐thaw‐sonication cycle was repeated three times. Samples were incubated at −20 °C for 1 h to precipitate proteins, centrifuged at 13 000 rpm for 15 min at 4°C, and 800 µL of supernatant was collected, dried under vacuum at low temperature, and stored at −80°C until LC‐MS analysis.

In vivo, mice were infected with *E. multilocularis* metacestodes for three months prior to treatment. The mice received daily intraperitoneal injections of 150 mg/kg of NAC in sterile PBS for 7 consecutive days, with a 24‐h injection interval. Metacestode tissues were harvested from the sacrificed mice at the following time points: Day 0 (pre‐treatment baseline), Day 1, Day 2, Day 4, and Day 7 after initiating NAC administration. Each experimental group and each time point included three independent biological replicate mice.

Immediately after euthanasia, the metacestodes were dissected from the liver, snap‐frozen in liquid nitrogen, and stored at −80°C until further processing. Tissue samples (20 mg each) were homogenized in 200 µL of ultrapure water using a mechanical homogenizer at 5 500 rpm for 20 s, repeated three times, under low‐temperature conditions. Then, 800 µL of extraction solvent (methanol: acetonitrile = 1:1, v/v) was added to each homogenate and vortexed for 30 s, followed by ice‐bath sonication for 10 min. The samples were then incubated at 5°C–20°C for 1 h to facilitate protein precipitation. After centrifugation at 13 000 rpm for 15 min at 4°C, 800 µL of the supernatant was collected and concentrated to dryness under vacuum at a low temperature. The dried extracts were stored at −80°C until LC‐MS analysis.

Metabolites related to energy metabolism (e.g., lactate, malate, TCA‐cycle intermediates, NADH/NADPH) were extracted from the raw data and compared across control and treatment groups. Enrichment analysis of differentially abundant metabolites was performed using the MetaboAnalyst web platform. Heatmaps visualizing metabolite expression profiles were generated with RStudio. All metabolomic analyses were performed in independent experimental trials.

### 4.6 Identification and Cloning of EmPDK and EmPDH E1α Gene

Published sequences of PDK and PDH E1α of the human, mouse, *Drosophila*, *Xenopus*, zebrafish, and *C. elegans* were used as queries to BLAST the *E. multilocularis* genome database available at Wormbase database. EmuJ_000136800 and EmuJ_000590700 was identified as the homologs EmPDK and EmPDHE1α respectively and its full coding sequences were amplified from the cDNA preparations as described previously (Brehm et al., 2000) with custom‐designed specific primers listed in Table S3. The analysis of three‐dimensional structure was generated using the SWISS‐MODEL online platform. All modeling parameters were set to default values based on the automated homology modeling pipeline. Gene identification, PCR cloning, and structural prediction analyses were performed in at least three independent biological replicates.

### 4.7 EmPDK and EmPDH E1α Polyclonal Antibody Preparation

EmPDK and EmPDH E1α fragments were inserted into the prokaryotic vector pet‐22b to construct the recombinant plasmids. After full sequence verification, the correctly constructed plasmids were transformed into Escherichia coli BL21 (DE3) competent cells for recombinant protein expression. Bacterial cultures were induced with 0.1 mM IPTG at 20°C with continuous shaking at 160 rpm for 14 h. Subsequently, bacterial pellets were collected and lysed by ultrasonication under low‐temperature conditions. His‐tagged recombinant EmPDK and EmPDH E1α proteins were purified using the Biyuntian His Label purification Kit purified protein (P2229s, China) following the manufacturer's protocols, and the purity of eluted proteins was confirmed via SDS‐PAGE electrophoresis. Purified proteins were concentrated using 50 mL ultrafiltration tubes to meet the antibody preparation criteria, with a qualified protein concentration ≥ 1 mg/mL and total volume ≥ 2 mL. Polyclonal antibodies against EmPDK and EmPDH E1α were prepared by immunizing New Zealand rabbits with subcutaneous multipoint injection. Upon completion of the standardized 4‐week immunization procedure, partial rabbit serum was collected for quality assessment, including ELISA‐based titer detection and Western blot‐based antigen‐specificity verification. Only serum samples with robust antibody titers and specific immunoreactive bands matching the target protein molecular weight were deemed qualified. Whole blood was then collected from immunized rabbits and centrifuged at 3000 g for 15 min at room temperature, and the supernatant serum was harvested as the final polyclonal antibody for subsequent experiments. Both EmPDK and EmPDH E1α polyclonal antibodies were used for Western blot analysis at a unified dilution ratio of 1:500. All prokaryotic expression and protein purification experiments were performed in three independent biological replicates.

### 4.8 Western Blot

Western blotting was performed in accordance with standard procedures. Briefly, cells were lysed for 5 min in ice‐cold lysis buffer supplemented with PMSF and phosphatase and protease inhibitors. After centrifugation, protein samples with equal amounts of protein were separated by sodium dodecyl sulphate–polyacrylamide gel electrophoresis. After proteins had been transferred to polyvinylidene difluoride membranes using a constant current of 300 mA for 150 min. After transfer, the membranes were blocked with blocking buffer at room temperature for 2 h to eliminate non‑specific binding. Subsequently, the membranes were incubated with primary antibodies (4°C, overnight), including anti‐EmHif1α (1:1000), anti‐EmPDK (1:500), anti‐EmPDH E1α (1:500), anti‐p‐PDHA1 (1:2000, Ab92696, Abcam), anti‐EmLDHA (1:2000, #3582, CST), and anti‐α‐tublin (1:5000, A6830, ABclonal). After thorough washing, the membranes were incubated with horseradish peroxidase‐conjugated secondary antibody (1:10000, ABclonal) at room temperature for 1 h, then visualised using a chemiluminescent reagent. The signal density for each band was quantified using Image Lab software.

### 4.9 SiRNA Preparation and Delivery to *E. multilocularis* Protoscoleces and Vesicle Cells

The EmPDK RNAi target sequence was designed and synthesized by Ribobio (Guangzhou, China). The three sirna sequences targeting EmHIF1α and EmPDK are listed in Table S3.

We established four siRNA groups: a negative control siRNA group (siNC) and three EmPDK siRNA‐treated groups. Electroporation was used to deliver siRNA into protoscoleces in vitro according to a previously established protocol (Spiliotis et al., 2010). In brief, 2000 protoscoleces and 2 × 10^6 vesicle cells were washed three times with RNAi electroporation buffer (120 mM trehalose, 20 mM HEPES, 1 mM myo‐inositol, 1 mM KCl, 1 mM MgCl2, 1 mM K2HPO4, 0.4 mM KH2PO4 and 1 mM gluthatione, pH 6.9) and then resuspended in 100 µL electroporation buffer containing FAM‐labelled control siRNA to a final concentration of 3 µm in a 1‐mm electroporation cuvette. Electroporation was performed pulses once with 125 V for 20 ms by Gene Pulser II (Bio‐Rad, United States). After incubation at 37°C for 10 min, 2 mL culture medium was added, and the protoscoleces and vesicle cells were transferred to 12‐well plates for an additional 36–40 h of incubation at 37°C in 5% CO2 in the dark. Then, some protoscoleces and vesicle cells were used to collect protein samples, some protoscoleces and vesicle cells were used to collect Real‐time quantitative PCR samples.

### 4.10 Real‐Time Quantitative PCR

Total vesicle RNA was collected by RNeasy Mini Kit (ABclonal, China). RNA was converted to cDNA by PrimeScript RT reagent Kit with gDNA Eraser (ABclonal, China). Real‐time PCR was carried out using qPCR SYBR Green Master Mix (ABclonal, China) in LightCycler 96 (Roche, Germany). Primer sequences for target genes and the endogenous reference gene are provided in Table S3. The Elp gene was used for transcript normalization (Brehm et al., 2003). Relative gene expression levels were quantified via the 2^(−ΔΔCt) method. All qRT‐PCR assays were performed with three independent biological replicates for each sample.

### 4.11 Lactic Acid Content Detection

Lactic acid content detection was performed according to the standard protocol of the lactate assay kit (ab65331, USA). Specific steps are as follows: Cells (2 × 10^6^ cells) or tissue (10 mg) were washed in pre‐chilled PBS. Add 4 volumes of lactate assay buffer (approximately 200 µL) to cells and briefly pipette to homogenize. For tissue, add 4–6 volumes of lactate assay buffer and homogenize on ice. Centrifuge at high speed at 4°C for 2–5 min to remove insoluble material. Transfer the supernatant to a new centrifuge tube, maintaining all steps on ice. Prepare standards and reaction mixture according to the manual. Add 50 µL of reaction mixture to each standard (50 µL) and sample (50 µL) well and mix thoroughly (3 replicate wells per sample). Incubate at room temperature for 30 min. Read the optical density (OD) at 450 nm using a Tecan Spark microplate reader (Switzerland). Calculate the final lactate concentration according to the method described in the manual. All experiments were performed in three independent biological replicates. Based on the relative rates of lactate production and secretion across different cell types, intracellular lactate concentrations can range from approximately 0.5 mm to 15–30 mm.

### 4.12 In Vitro PDK Kinase Activity Assay

EmPDK‐mediated phosphorylation of EmPDH E1α at the Ser293 site was validated using an in vitro targeted peptide kinase assay with the Phosphate Sensor Assay Kit (Beyotime, S0192S). Custom synthetic peptides (TYRYFGH*S*MSDPG) spanning the Ser293 residue of EmPDHE1α were utilized as substrates. All kinase reactions were performed in a 20 µL system containing kinase buffer (25 mM HEPES, pH 7.4, 5 mM MgCl_2_, 1 mM DTT), recombinant EmPDK (100–200 ng), and 1.5 µg substrate. The reaction was initiated by adding ATP (final concentration: 80 µm) and incubated at 30°C for 30–60 min. Reactions were terminated, and the samples were diluted in 1× Assay Buffer (from the kit) to ensure the phosphate concentration fell within the linear detection range (approximately 200 to 2 µM). For detection, 10 µL of the diluted sample was mixed with 10 µL of 10 µM Phosphate Sensor (prepared in 1× Assay Buffer) in a black 384‐well plate. A standard curve was generated in parallel using inorganic phosphate standards (0–2 mM) diluted in 1× Assay Buffer. Fluorescence was measured with a microplate reader at 25°C using excitation/emission wavelengths of 430/450 nm. EmPDK activity was calculated as phosphate production per minute per microgram of protein based on the standard curve. For inhibitor studies, DCA was included in the reaction mixture at indicated concentrations. Statistical analysis was performed using data from three independent biological experiments.

### 4.13 Seahorse XFe96 Metabolic Flux Analysis

XFe96 plates were pre‐coated with 25 µL of 22.4 µg/mL Cell‐Tak per well, washed twice with sterile water, and equilibrated to room temperature. Primary cells isolated from *E. multilocularis* metacestode vesicles were centrifuged at 200 × g for 5 min, resuspended in pre‐warmed assay medium, and seeded at 1 × 10^4^ cells per well in 50 µL medium; blank wells contained medium only. Plates were centrifuged at 200 × g (zero brake) for 1 min and incubated at 37°C in a CO_2_‐free incubator for 25–30 min to facilitate cell attachment. Each well was supplemented with 130 µL pre‐warmed assay medium, followed by 15–25 min of equilibration. All post‐centrifugation operations were finished within 60 min to ensure metabolic stability. OCR measurement adopted sequential injections of 20 µL oligomycin (1.5 µM), 22 µL FCCP (0.5 µM), and 25 µL rotenone/antimycin A (0.5 µM) via ports A, B and C, respectively. ECAR assays were performed with identical cell preparation procedures, with sequential injections of 20 µL glucose (10 mM), 22 µL oligomycin (1 µM), and 25 µL 2‐DG (50 mM). Dynamic OCR and ECAR curves were recorded using the Seahorse XFe96 analyzer. All assays were performed with three technical replicates per group.

### 4.14 Dual‐Luciferase Reporter Assay

The *EmPDK* 1500 bp promoter region upstream of the identified transcription start site was analyzed, and two high‐score HREs (821–826 and 1021–1026; core ACGTG) were predicted using JASPAR, with the latter representing a high‐confidence HIF1α‐binding motif. A 930 bp fragment encompassing both HREs was cloned into pGL3‐Basic, and a mutant reporter was similarly constructed by mutating the high‐confidence HRE to AAAAAA via site‐directed mutagenesis. For dual‐luciferase reporter assays, adherent human 293T cells in 6 cm dish were co‐transfected with 4 µg pGL3‐basic‐EmPDK promoter reporter construct, 2 µg pcDNA3.3‐N‐Myc‐EmHIF1α or empty pcDNA3.3‐N‐Myc control vector, and 400 ng pRL‐TK normalization plasmid, and incubated for 24 h. Cells were lysed in 500 µL Dual‐Lumi lysis buffer after medium removal, and clarified supernatants were collected via centrifugation at 10 000–15 000 × g for 3–5 min. Firefly luciferase reagent and Renilla assay buffer were equilibrated to ambient temperature, and 100× Renilla substrate was diluted 1:100 to prepare working solution. A total of 20 µL lysate supernatant was mixed with 100 µL firefly reagent, incubated for 5 min at ambient temperature, and firefly luminescence was measured using a multifunctional microplate reader; 100 µL Renilla working solution was then added to the same well for Renilla signal quantification. Relative EmPDK promoter activity was calculated as the ratio of firefly to Renilla luminescence values per well.

### 4.15 Data Analysis and Statistics

All statistical analyses were performed using SPSS (v24.0, SPSS Inc., Chicago, IL, USA). All data are presented as the mean ± standard deviation from at least three independent biological experiments. Statistical analyses were performed using raw ΔCt values derived from three independent biological replicates per experimental group. One‐way ANOVA was used to assess overall group differences, followed by Tukey's HSD post‐hoc test for pairwise comparisons. Adjusted *p < 0.05* was defined as statistically significant, with significance levels annotated as *p < 0.05, **p < 0.01, and ***p < 0.001.

For metabolomic profiling, orthogonal partial least squares discriminant analysis (OPLS‐DA) was used to calculate variable importance in projection (VIP) values, and metabolites with VIP > 1 were defined as core differential metabolites. Preliminary screening of differential metabolites adopted a platform‐default uncorrected threshold of *p* *< 0.1* without manual adjustment, employing this unadjusted threshold solely for exploratory candidate metabolite selection. Identified metabolites were further categorized by fold change to define upregulated and downregulated trends. False discovery rate (FDR) correction was applied only to KEGG pathway enrichment analysis to correct for multiple testing and ensure robust pathway‐level inferences, a standard dual‐tiered screening approach in metabolomics. For metabolite visualization and clustering, heatmaps of differential metabolites identified in exploratory screening were generated using the pheatmap package in R (v4.4.1). Metabolite expression data were log2‐transformed and row‐wise Z‐score normalized, and sample hierarchical clustering was performed based on Pearson correlation coefficients. The heatmap was annotated with metabolite names, with a color gradient from blue (low expression) to red (high expression) representing normalized Z‐scores.

## Author Contributions

**Yanhai Wang**: Writing – review and editing, conceptualization, funding acquisition, resources, project administration. **Ye Tian**: software, validation. **Yao Zhang**: methodology. **Huijuan Wang**: software, data curation, formal analysis, investigation, writing – original draft, validation, methodology. **Zhe Cheng**: conceptualization, writing – review and editing, funding acquisition, resources. **Zhijian Xu**: methodology.

## Funding

This research was supported by the National Natural Science Foundation of China (82272365 and 82572599), the NHC Key Laboratory of Echinococcosis Prevention and Control (no. 2025WZK20004), the National Parasitic Resources Center and the Ministry of Science and Technology Fund (NPRC‐2019‐194‐30). The funders had no role in study design, data collection and interpretation, or the decision to submit the work for publication.

## Conflicts of Interest

The authors declare no conflicts of interest.

## Supporting information




**Supporting File 1**: advs76829‐sup‐0001‐SuppMat.docx.

**Supporting File 2**: advs76829‐sup‐0002‐TableS1‐S3.zip.

## Data Availability

The data that supports the findings of this study are available in the supplementary material of this article.
