# Supplementary material for: Adaptation of Echinococcus Multilocularis to Oxidative Stress Depends on EmPDK‐Mediated Metabolic Reprogramming
Source: Adv Sci (Weinh). 2026 Aug 13;13(59):e76829. doi: 10.1002/advs.76829 (PMC13472117; doi:10.1002/advs.76829)
Supplement: Supplementary file 1 [file ADVS-13-e76829-s002.docx]

Supplementary Information:

**Adaptation of *Echinococcus multilocularis* to oxidative stress depends on EmPDK-mediated metabolic reprogramming**

*Huijuan Wang^1,2^, Zhijian Xu^1,2^, Ye Tian^,3^, Yao Zhang^1,2^, Zhe Cheng^1,2^*, Yanhai Wang^1,2^*.*

**Figure S1.**

**A, E.** OPLS-DA score plot of the control versus H₂O₂-treated groups and H₂O₂-treated groups versus co-treatment (H₂O₂ + NAC) groups for 4 h.

**B, F.** Model explanation (R2Y) and prediction (Q2) parameters for the control versus H₂O₂-treated groups and H₂O₂-treated groups versus co-treatment (H₂O₂ + NAC) groups for 4 h.

**C, G.** Volcano plot of control versus H₂O₂-treated groups and H₂O₂-treated groups versus co-treatment (H₂O₂ + NAC) groups for 4h.

**D, H.** Heatmap of differentially metabolites identified by exploratory screening.

**Figure S2.**

**A, E.** OPLS-DA score plot of the control versus NAC-treated groups and NAC-treated groups versus co-treatment (H₂O₂ + NAC) groups for 4 h.

**B, F.** Model explanation (R2Y) and prediction (Q2) parameters for the control versus NAC-treated groups and NAC-treated groups versus co-treatment (H₂O₂ + NAC) groups for 4 h.

**C, G.** Volcano plot of the control versus NAC-treated groups and NAC-treated groups versus co-treatment (H₂O₂ + NAC) groups for 4 h.

**D, H.** Heatmap of differentially metabolites identified by exploratory screening.

**I, J.** KEGG pathway enrichment based on exploratory differential metabolites for comparisons of control vs. NAC treatment and NAC treatment vs. H₂O₂ + NAC co-treatment at 4 h (red arrow: TCA cycle). Pathway significance was determined using FDR-corrected statistics.

**K-L.** Time-course profiles of OCR and ECAR were measured in *E. multilocularis* metacestode vesicles subjected to control, H₂O₂, NAC, and H₂O₂ + NAC treatments. Data are presented as mean ± SD (n=3 technical replicates per group).

**Figure S3.**

**A, D, G, J.** OPLS-DA score plots of *E. multilocularis* lesions from control versus NAC-treated (1,2,4, and 7 days post-treatment) infected mice.

**B, E, H, K.** Model explanation (R2Y) and prediction (Q2) parameters for the control versus NAC-treated (1, 2, 4, and 7 days) *E. multilocularis* lesions.

**C, F, I, L.** Volcano plots of control versus NAC-treated (1, 2, 4, and 7 days) *E. multilocularis* lesions.

**M-O.** KEGG pathway enrichment based on exploratory differential metabolites from *in vivo* metacestode lesions following NAC treatment for 1, 2, and 4 days (red arrow: TCA cycle). Pathway significance was FDR-corrected for multiple testing.

**Figure S4.**

**A.** Predicted three‑dimensional structures of *Homo sapiens* and *E. multilocularis* PDK proteins.

**B.** Predicted three-dimensional structures of *Homo sapiens* and *E. multilocularis* PDH E1α proteins. Models were generated using SWISS-Model.

**C.** Multiple sequence alignment of functional domains in PDHE1α across *E. multilocularis* and other indicated species.

**D.** Predicted binding pose of the inhibitor DCA within the EmPDK active site.

**E.** Purification profile of recombinant EmPDK expressed from the pET-22b vector.

**F.** Purification profile of recombinant EmPDHE1α expressed from the pET-22b vector. **G, I.** Titers of antisera raised against recombinant EmPDK and EmPDH E1α. x-axis: serum dilution; y‑axis: signal ratio between immune serum and pre‑immune serum. Above the red line is considered usable.

**H, J.** Immunoblot analysis of His‑tagged EmPDK (H) and EmPDHE1α(J) expression. (H) Lane 1: pet-22b-EmPDK bacteria without IPTG induction; Lanes 2–3: IPTG-induced lysate and concentrated protein. (J) Lane 1: blank control with no recombinant EmPDHE1α and no IPTG; Lanes 2–3: IPTG-induced lysate and concentrated protein.

**K.** Immunoblot analysis of p‑EmPDHE1α in metacestode vesicle lysates after phosphatase treatment.

**L.** Immunoblot analysis of p‑EmPDHE1α and total Flag‑tagged protein in 293T cells transiently expressing wild‑type pcDNA3.3‑Flag‑EmPDHE1α or a phosphorylation‑site mutant (S293A).

**Figure S5.**

**A.** Immunofluorescence staining of metacestode vesicles treated with DCA for 4 h. DAPI (blue), EdU (red), and anti‑p‑EmPDHE1α (green). White arrows indicate EdU^+^/p‑EmPDH^+^ double‑positive cells.

**B, G.** Quantification of EdU^+^, p‑EmPDH^+^, and EdU^+^/p‑EmPDH^+^ double‑positive cells per 1000 cells in metacestode vesicles treated with DCA for 4 h (B), 48 h (G).

**C-F and H-K.** Quantification of EdU^+^/ p‑EmPDH^+^ cells following DCA treatment for 4 h (C-F) and 48 h (H-K).

**L-O.** Quantification of EdU^+^/ p‑EmPDH^+^ cells in liver lesion from mice with treated DCA for 2 weeks.

Data in **(B–K, L–O)** are presented as mean ± SEM; B–K: single metacestode vesicle (n=8 biological replicate vesicles); L–O: individual infected mouse (n=7 biological replicates); one-way ANOVA with Tukey’s HSD post-hoc test. **p < 0.05, **p < 0.01, ***p < 0.001,* ns = not significant.

**Figure S6.**

**A.** OPLS-DA score plot of the control versus DCA-treated groups for 4 h.

**B.** Model explanation (R^2^Y) and prediction (Q^2^) parameters for the control versus DCA-treated groups for 4 h.

**C.** Volcano plot of control versus DCA-treated groups for 4 h.

**D.** Heatmap of exploratory differential metabolites.

**E–G.** KEGG pathway enrichment analysis using exploratory differential metabolites in metacestodes after 4h treatment. Pathways were compared between control and DCA (E), H₂O₂ (F), and co‑treatment with H₂O₂ + DCA (G). Red arrows: TCA cycle; orange arrows: pyruvate metabolism and glycolysis. Pathway significance was FDR-corrected for multiple testing.

**Figure S7.**

**A.** Dual-luciferase reporter analysis in 293T cells. Ctrl: empty vector; 1: pGL3-basic-EmPDK promoter + pcDNA3.3-N-Myc + pRL-TK; 2: pGL3-basic-EmPDK promoter + pcDNA3.3-N-Myc-EmHIF1α + pRL-TK; 3: HRE-mutant EmPDK promoter reporter + pcDNA3.3-N-Myc + pRL-TK; 4: HRE-mutant EmPDK promoter reporter + pcDNA3.3-N-Myc-EmHIF1α + pRL-TK. Relative firefly/Renilla luciferase activity was normalized to empty vector control. Individual well seeded with 293T cells; n = 3 biological replicates, three technical replicates per condition. Five-group comparisons analyzed by one-way ANOVA with Tukey’s HSD post-hoc test. Data are presented as mean ± SD; **p < 0.05, **p < 0.01, ***p < 0.001*, ns = not significant.

**B.** Schematic of the EmPDK promoter containing two predicted HRE elements upstream of TSS. The core ACGTG motif of the distal HRE1 (1021–1026) was replaced with AAAAA to construct the mutant reporter plasmid. HRE, hypoxia response element; TSS, transcription start site.
