# Supplementary material for: Adaptation of Echinococcus Multilocularis to Oxidative Stress Depends on EmPDK‐Mediated Metabolic Reprogramming
Source: Adv Sci (Weinh). 2026 Aug 13;13(59):e76829. doi: 10.1002/advs.76829 (PMC13472117; doi:10.1002/advs.76829)
Supplement: Supplementary file 2 [file ADVS-13-e76829-s001.zip › SupplementaryTableInformation.docx]

**Supplementary Information:**

**Adaptation of *Echinococcus multilocularis* to oxidative stress depends on EmPDK-mediated metabolic reprogramming**

*Huijuan Wang^1,2^, Zhijian Xu^1,2^, Ye Tian^,3^, Yao Zhang^1,2^, Zhe Cheng^1,2^*, Yanhai Wang^1,2^*.*

**Table S1.1 Identification of differential metabolites using mass spectrometry**

**Column 1.** Component name: Metabolite name

**Column 2.** HMDB: Human Metabolome Database ID: Used to uniquely identify the metabolite.

**Column 3-8.** Ctrl-1~3 & H_2_O_2_-1~3: This shows the metabolite's peak area in the three replicate samples of each group. This represents the metabolite's relative abundance.

**Column 9.** VIP (Variable Importance in Projection): A key metric that quantifies the contribution of each metabolite to distinguishing between the control (Ctrl) groups and the H₂O₂-treated groups. A VIP value greater than 1 indicates that the metabolite exhibits significant differences or high discriminative power between the groups.

**Table S1.2 Identification of differential metabolites using mass spectrometry**

**Column 1.** Component name: Metabolite name

**Column 2.** HMDB: Human Metabolome Database ID: Used to uniquely identify the metabolite.

**Column 3-8.** H_2_O_2_-1~3 & H_2_O_2_+NAC-1~3: This shows the metabolite's peak area in the three replicate samples of each group. This represents the metabolite's relative abundance.

**Column 9.** VIP (Variable Importance in Projection): A key metric that quantifies the contribution of each metabolite to distinguishing between the H_2_O_2_-treated groups and the H₂O₂+NAC-treated groups. A VIP value greater than 1 indicates that the metabolite exhibits significant differences or high discriminative power between the groups.

**Table S1.3 Identification of differential metabolites using mass spectrometry**

**Column 1.** Component name: Metabolite name

**Column 2.** HMDB: Human Metabolome Database ID: Used to uniquely identify the metabolite.

**Column 3-8.** Ctrl-1~3 & NAC-1~3: This shows the metabolite's peak area in the three replicate samples of each group. This represents the metabolite's relative abundance.

**Column 9.** VIP (Variable Importance in Projection): A key metric that quantifies the contribution of each metabolite to distinguishing between the control (Ctrl) groups and the NAC-treated groups. A VIP value greater than 1 indicates that the metabolite exhibits significant differences or high discriminative power between the groups.

**Table S1.4 Identification of differential metabolites using mass spectrometry**

**Column 1.** Component name: Metabolite name

**Column 2.** HMDB: Human Metabolome Database ID: Used to uniquely identify the metabolite.

**Column 3-8.** NAC-1~3 & H_2_O_2_+NAC -1~3: This shows the metabolite's peak area in the three replicate samples of each group. This represents the metabolite's relative abundance.

**Column 9.** VIP (Variable Importance in Projection): A key metric that quantifies the contribution of each metabolite to distinguishing between the NAC-treated groups and the H_2_O_2_+NAC-treated groups. A VIP value greater than 1 indicates that the metabolite exhibits significant differences or high discriminative power between the groups.

**Table S1.5 Identification of differential metabolites using mass spectrometry**

**Column 1, 11, 21, 31.** Component name: Metabolite name

**Column 2, 12, 22, 32.** HMDB: Human Metabolome Database ID: Used to uniquely identify the metabolite.

**Column 3-8.** Ctrl-1~3 & 1 day-1~3: This shows the metabolite's peak area of *E. multilocularis* in the three mice of each group. This represents the metabolite's relative abundance.

**Column 13-18.** Ctrl-1~3 & 2 day-1~3: This shows the metabolite's peak area of *E. multilocularis* in the three mice of each group. This represents the metabolite's relative abundance.

**Column 23-28.** Ctrl-1~3 & 4 day-1~3: This shows the metabolite's peak area of *E. multilocularis* in the three mice of each group. This represents the metabolite's relative abundance.

**Column 33-38.** Ctrl-1~3 & 7 day-1~3: This shows the metabolite's peak area of *E. multilocularis* in the three mice of each group. This represents the metabolite's relative abundance.

**Column 9, 19, 29, 39.** VIP (Variable Importance in Projection): A key metric that quantifies the contribution of each metabolite to distinguishing between the control (Ctrl) groups and the NAC (1 day, 2 days, 4 days, 7days)-treated groups. A VIP value greater than 1 indicates that the metabolite exhibits significant differences or high discriminative power between the groups.

**Table S1.6 Identification of differential metabolites using mass spectrometry**

**Column 1, 11, 21.** Component name: Metabolite name

**Column 2, 12, 22.** HMDB: Human Metabolome Database ID: Used to uniquely identify the metabolite.

**Column 3-8.** Ctrl-1~3 & H_2_O_2_-1~3: This shows the metabolite's peak area in the three replicate samples of each group. This represents the metabolite's relative abundance.

**Column 13-18.** Ctrl-1~3 & DCA-1~3: This shows the metabolite's peak area in the three replicate samples of each group. This represents the metabolite's relative abundance.

**Column 23-28.** Ctrl-1~3 & DCA+H_2_O_2_-1~3: This shows the metabolite's peak area in the three replicate samples of each group. This represents the metabolite's relative abundance.

**Column 9, 19, 29.** VIP (Variable Importance in Projection): A key metric that quantifies the contribution of each metabolite to distinguishing between the control (Ctrl) groups and the (H₂O₂, DCA, DCA+H_2_O_2_)-treated groups. A VIP value greater than 1 indicates that the metabolite exhibits significant differences or high discriminative power between the groups.

**Table S2. KEGG pathway enrichment results**

**Table S2.1.** KEGG pathway enrichment results from mass spectrometry analysis of the control and H_2_O_2_-treated groups

**Table S2.2.** KEGG pathway enrichment results from mass spectrometry analysis of the H_2_O_2_-treated groups and H_2_O_2_+NAC-treated groups

**Table S2.3.** KEGG pathway enrichment results from mass spectrometry analysis of the control and NAC-treated groups

**Table S2.4.** KEGG pathway enrichment results from mass spectrometry analysis of the NAC-treated groups and H_2_O_2_+NAC-treated groups

**Table S2.5.** KEGG pathway enrichment results from mass spectrometry analysis of the control and NAC (1 day, 2 days, 4 days, 7 days)-treated groups

**Table S2.6.** KEGG pathway enrichment results from mass spectrometry analysis of the control and DCA-treated groups

**Table S2.7.** KEGG pathway enrichment results from mass spectrometry analysis of the control and H_2_O_2_-treated groups, the control and H_2_O_2_+DCA-treated groups

**Table S2.8.** KEGG enrichment analysis of differential metabolites identified via the Figure 4K workflow

**Column 1.** KEGG enriched pathway terms

**Column 2.** Total: The total number of differentially expressed metabolites included in this KEGG pathway.

**Column 3.** Expected: The 'expected' number of differentially expressed metabolites, calculated based on a hypergeometric distribution.

**Column 4.** Hits: The number of differentially expressed metabolites that were actually observed in this KEGG pathway.

**Column 5.** Raw p: The smaller the p-value, the greater the likelihood that the pathway is enriched with differentially expressed metabolites.

**Column 6.** Holm p (Holm-Bonferroni corrected p-value): This adjusts the significance level. The smaller this value, the higher the probability that the pathway remains significant.

**Column 7.** FDR (False Discovery Rate): This represents the proportion of pathways that are not actually significant among all pathways deemed 'significant'. A lower FDR is better.

**Table S3. Primer sequences used in this study**

The table lists the oligonucleotide primers designed for gene cloning, quantitative real-time PCR (qPCR), and mutant verification. F: forward primer; R: reverse primer. Gene symbols and their corresponding full names are as follows: EmPDK: *Echinococcus multilocularis* pyruvate dehydrogenase kinase; EmHIF1α: *E. multilocularis* hypoxia-inducible factor 1 alpha; EmPDHA1: *E. multilocularis* pyruvate dehydrogenase E1α subunit; EmLDHA: *E. multilocularis* lactate dehydrogenase A. All primers were synthesized by Bioray Biotech (Xiamen, China). The sequences are listed in the 5’ to 3’ orientation.
